# On the Variations in the Weight of Prisoners Submitted to the Penitentiary System

**Published:** 1846-01

**Authors:** 


Art. VI.—*Notice sur les Variations du Poids des Prisonniers soumis au Régime Pénitentiaire.* Par le Docteur Marc d'Espine, Médecin des Prisons et Membre du Conseil de Santé du Canton de Genève, &c. &c.

*On the Variations in the Weight of Prisoners submitted to the Penitentiary System.* By Dr. Marc d'Espine, &c. &c. Extracted from 'The Annales d'Hygiène publique et de Médecine légale.' Vol. xxxii, Part I.

We refer our readers to this essay for a species of information which is not to be found elsewhere. The second Report of the Prisons of England contains, it is true, a statement of the weight of prisoners on their entrance into and discharge from the house of correction at Devizes; but M. Marc d'Espine shows that these results are wanting in accuracy, from defective arrangement of the facts on which they are founded. The subject is well reasoned out in his own essay, though the facts are scarcely sufficiently numerous. The system pursued is a modification of the American system. The prisoners labour in common and in silence during the day, and occupy separate cells at night. The peculiarity is one well worthy of imitation. The prisoners are divided into four classes, and a promotion from the worst to the next one above it is attended with some slight indulgence, which offers a motive to good conduct and holds out a hope of improvement. The inmates are not, as in the worst of our penal settlements, cut off from all hope. The effect of this wise arrangement displays itself in the improved health of those who have reached the two upper classes, as tested by an increase of weight. On the average of all the prisoners there is no material gain or loss of weight; but those who are placed in the most favorable circumstances have also the best health, and are found, on the average, to increase in weight. The following are the author's own conclusions:—1. That the number of prisoners emaciated by the penitentiary system is a little larger than that of those who have increased in weight. 2. That this difference, less than we should imagine, *à priori*, is remarkable only in the divisions in which the regimen is most severe. 3. That the loss of flesh does not occur in the first month of confinement, but much later. 4. That summer or winter, cold or heat, do not seem to exert a special influence on the degree of emaciation.

